# Dynamic air/liquid pockets for guiding microscale flow

**DOI:** 10.1038/s41467-018-03194-z

**Published:** 2018-02-21

**Authors:** Xu Hou, Jianyu Li, Alexander B. Tesler, Yuxing Yao, Miao Wang, Lingli Min, Zhizhi Sheng, Joanna Aizenberg

**Affiliations:** 1 Research Institute for Soft Matter and Biomimetics, College of Physical Science and Technology, Xiamen University, Xiamen, 361005 China; 2 College of Chemistry and Chemical Engineering, Collaborative Innovation Center of Chemistry for Energy Materials, State Key Laboratory of Physical Chemistry of Solid Surface, Xiamen University, Xiamen, 361005 China; 3 John A. Paulson School of Engineering and Applied Sciences and Wyss Institute for Biologically Inspired Engineering, Harvard University, Cambridge, MA 02138 USA; 4 Department of Mechanical Engineering, McGill University, Montreal, QC H3A 0G4 Canada; 5 Department of Chemistry & Chemical Biology, Harvard University, Cambridge, MA 02138 USA

## Abstract

Microscale flows of fluids are mainly guided either by solid matrices or by liquid–liquid interfaces. However, the solid matrices are plagued with persistent fouling problems, while liquid–liquid interfaces are limited to low-pressure applications. Here we report a dynamic liquid/solid/gas material containing both air and liquid pockets, which are formed by partially infiltrating a porous matrix with a functional liquid. Using detailed theoretical and experimental data, we show that the distribution of the air- and liquid-filled pores is responsive to pressure and enables the formation and instantaneous recovery of stable liquid–liquid interfaces that sustain a wide range of pressures and prevent channel contamination. This adaptive design is demonstrated for polymeric materials and extended to metal-based systems that can achieve unmatched mechanical and thermal stability. Our platform with its unique adaptive pressure and antifouling capabilities may offer potential solutions to flow control in microfluidics, medical devices, microscale synthesis, and biological assays.

## Abstract (web summary)

Fouling of solid surfaces is a problem when designing microchannel systems for applications such as bioassays and drug delivery. Here Hou et al. propose a way to overcome this issue by controlling fluid flow by means of an immiscible functional liquid partly infiltrated in a porous solid matrix.

## Introduction

Controlling microscale flows of gas and liquid is essential in design, fabrication, and implementation of microchannel systems for applications ranging from bioassays, micro-valves, chemical and biological sensing, drug delivery, and environmental analysis to production of high-value fluids and petrochemicals^1–11^. The flow is mainly guided either by solid matrices or by liquid–liquid interfaces. Although solid matrices are ubiquitous in most devices that are designed to regulate microscale flows, their application is plagued with the problem of fouling when contaminating components (notably large organic and biological molecules) present in the transport fluid irreversibly adhere to the solid surfaces^12–14^. Strategies to combat fouling include either the direct use of low-surface-energy matrix materials (such as polydimethylsiloxane (PDMS) and fluoropolymers)^13^ or chemical modification of high-surface-energy solids (such as glass)^14^; however, these strategies often provide only transient or limited antifouling functions, and a long-term solution remains elusive. Guiding flows with liquid–liquid interfaces can eliminate the fouling on solid surfaces, but the liquid–liquid interfaces resulted from surface-directed coating are only stable for low pressures on the order of 1 kPa, which in turn strictly limits their applications^2,3^.

Here we introduce a dynamic, hybrid liquid/solid/gas material system that guides microscale flows with high-performance antifouling, self-recovery, and liquid-gating properties. The design of such a dynamic system allows us to achieve extraordinary function that is impossible with conventional materials^15,16^. Our idea is inspired by the structure of the liquid linings in gastrointestinal tract that separate epithelium from the highly corrosive, low-pH environment in the stomach or from the waste flow in the colon^17^. These structures have evolved into micro-porous surfaces composed of cavities filled with protective mucus that forms a stable liquid defense layer dynamically supplied to the interface. Following this rationale, we design an adaptive air/liquid pocket transport system (ADAPTS) for guiding microscale flows, which consists of an interconnected porous matrix partially infiltrated with a functional liquid and a microchannel constructed inside. Importantly, the functional liquid not only protects the solid matrix from the fouling similar to the mucus lining in biological systems, it can also reversibly enter or exit the microchannel by diffusion from or wicking into the porous matrix, which can be further controlled by external stimuli (for example, pressure).

## Results

### Design of the adaptive air/liquid pocket transport system

**Fig.1:**
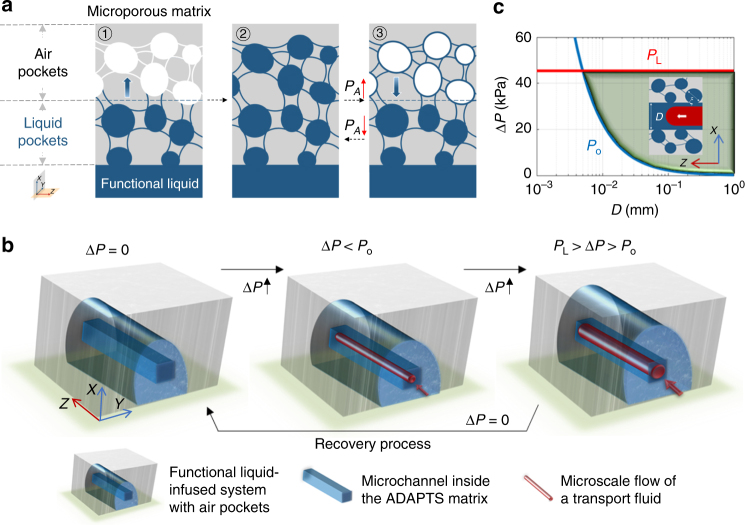
Design of the adaptive air/liquid pocket transport system (ADAPTS). **a** Schematic of ADAPTS, consisting of a porous matrix partially infiltrated with a functional liquid (shown in blue). The distribution of the functional liquid-filled pores (liquid pockets) and air-filled pores (air pockets) changes with the pressure in the air pocket (*P*_A_). **b** ADAPTS guides the microscale flow (shown in red) in the square microchannel (shown in dark blue). The transport liquid initiates a flow path when the applied pressure (Δ*P*) is beyond a threshold pressure (*P*_o_) and is confined inside the microchannel under a critical pressure (*P*_L_). After removal of the pressure, the functional liquid refills the microchannel, and ADAPTS recovers immediately to the original state. **c** Diagram showing the two critical pressures (*P*_o_, *P*_L_) as a function of the channel size *D*. The operating range of ADAPTS is marked in light green. The inset shows the invasion of the transport liquid into ADAPTS with average pore size *ξ*

Inspired by the structure of the liquid linings in gastrointestinal tract (Fig. 1a and Supplementary Fig. 1), functional ADAPTS are designed following two criteria: the porous matrix should be preferentially wetted by the functional liquid but not by the transport liquid in the microchannel; the microchannel should be initially fully filled with the functional liquid. Microchannels can be constructed inside the ADAPTS matrix with the laser cutting technique and be fabricated practically into any size, shape, and dimension (Supplementary Figs 2 and 3). To satisfy the first criterion, we chose Krytox®103 as the functional liquid and polytetrafluoroethylene (PTFE) as the porous matrix^18^ in our initial experiments. Driven by the capillary pressure, the functional liquid infiltrates the air pockets from the microchannel spontaneously (Fig. 1b). To satisfy the second criterion, one could fill the matrix completely with the functional liquid and then balance the capillary pressure by controlling the pressure in the air pockets (*P*_A_). When the air pocket pressure overcomes the capillary pressure, the liquid pockets transform into the air pockets with surfaces wetted by the functional liquid (Fig. 1b).

As discussed below, this design enables intrinsic liquid-gating, instantaneous self-recovery, and ability to function at high pressure inside the microchannel. When a transport fluid enters the microchannel initially filled with the functional liquid, it displaces the functional liquid to form a cylindrical pathway in the microchannel (Fig. 1c). The liquid–liquid displacement will take place only if the applied pressure (Δ*P*) is equal to or greater than a threshold pressure (*P*_o_), which depends on the size of microchannel *D* and the interfacial tension between the two liquids ${\mathbf{\gamma }}_{{\mathrm{FT}}}$ via $4{\mathbf{\gamma }}_{{\mathrm{FT}}}/D$, where the subscripts F and T denote the functional and transport liquid, respectively (Supplementary Table 1). The transport liquid will invade the porous matrix when the pressure surpasses another critical pressure, *P*_L_ = $4{\mathbf{\gamma }}_{{\mathrm{FT}}}/\xi$, where $\xi$ is the average size of the air/liquid pocket (e.g., average pore size). The critical pressure *P*_L_ defines the upper boundary of the operating pressure. If the applied pressure is removed, the functional liquid will refill the microchannel, and the system restores its original state instantaneously (Supplementary Fig. 4). The two critical pressures (*P*_o_, *P*_L_) can be rationally tuned by controlling the pore size distribution, interfacial tensions, and channel sizes. As an example, Fig. 1c presents a diagram for designing and operating the ADAPTS with an average pore size of 5 μm, for which the critical pressure *P*_L_ is around 45 kPa, nearly two orders of magnitude higher than the pressures achievable in simple liquid–liquid interfaces^2,3^.

### Dynamic control of air/liquid pockets

**Fig. 2:**
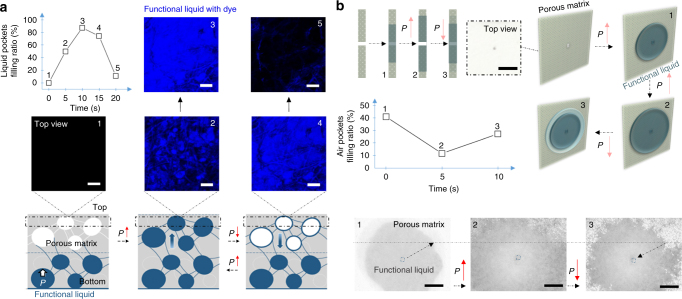
Pressure-induced redistribution of air and liquid pockets. **a** Confocal microscopic studies. Functional liquid is silicone oil with dye DFSB-K175. The liquid pockets' filling ratio (determined by measuring the fluorescence intensity in the air pockets) dynamically changes with the applied pressure over time. Confocal images of air pockets in their original unfilled state (image 1), during their transition into liquid pockets by pressure-induced diffusion (image 2→3), and the recovery of the initial air-filled state after the removal of pressure (image 4→5). The corresponding schematics show the location of the images. Scale bar is 100 μm. **b** Optical microscopy studies of dynamic distribution of air and liquid pockets under applied pressure. Functional liquid is Krytox®103. The cross-section view of the ADAPTS microchannel to illustrate the dynamic change of liquid pockets’ distribution, and schematic of the changes of liquid pockets’ distribution under varying pressures. The corresponding experimental images show the top view of the air/liquid pockets’ distribution. Scale bar is 2 mm. The graph shows the air pockets' filling ratio and its change with the applied pressure over time (determined by measuring the area occupied by the air pockets that appear translucent in optical images)

Experimental observations of this dynamic, reversible redistribution of the air and liquid pockets inside the porous matrix are shown in Fig. 2. As seen from the confocal microscopic images in Fig. 2a, the porous matrix is partially filled with the functional liquid; the dye-labeled functional liquid moves into the porous matrix with increasing applied pressure, filling the available air pockets over time; as the pressure is removed, the liquid is displaced from the filled pores into the channel and the initial distribution of the air and liquid pockets is restored. Optical images in Fig. 2b illustrate this effect on a macroscopic scale, providing further evidence of the dynamic nature of the ADAPTS matrix in which the transition between the liquid and air pockets is reversible and responsive to external stimuli. The presence of the air pockets offers therefore an important, unique replenishment mechanism—the ability to store and release the displaced functional liquid.

### Dynamic liquid–liquid interface

**Fig. 3:**
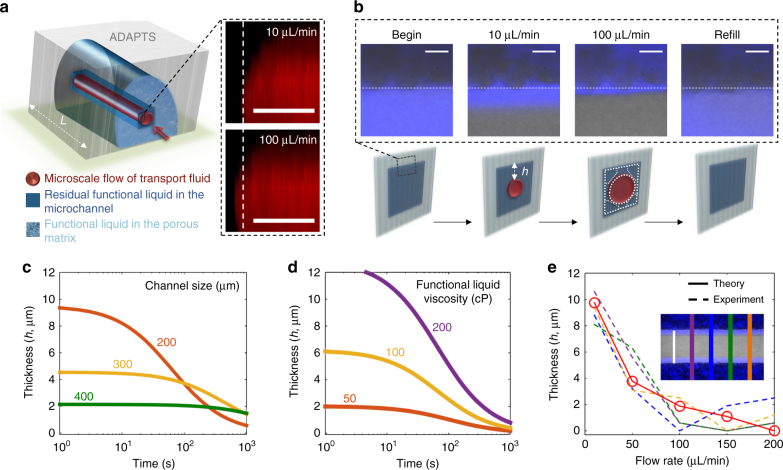
Dynamic liquid–liquid interfaces in the microchannel. **a** Liquid transport inside the microchannel of ADAPTS matrix. Confocal images of the transport liquid (Rhodamine B aqueous solution, red) show the cylindrical flow profile in a square microchannel. The cross-section area of the flow varies with the flow rate. Scale bar is 50 µm. **b** Remaining functional liquid in the microchannel. Confocal images and corresponding schematics show the stable functional liquid film (silicone oil lubricant with dye DFSB-K175, blue) at the channel wall for two different flow rates. Scale bar is 20 μm. After removal of the applied pressure, the functional liquid refills the microchannels. The white dashed lines are added as a reference. **c**, **d** Theoretical modeling of the average thickness, *h*, of the remaining functional liquid as a function of the flow time for different channel sizes (**c**) and viscosities of the functional liquid (**d**). **e** Comparison of the experimental and theoretical values for the average thickness of the remaining functional liquid on the channel wall as a function of the flow rate. The inset shows the confocal image of the microchannel with the functional liquid (in blue), in which the solid lines indicate the measuring positions. Scale bar is 200 μm (white line in the inset). The flow rate of the transport liquid is 10 µL/min

In contrast to macroscopic channels with diameters of a few millimeters, in which the surface-held functional liquid gets depleted (often completely) by the transport fluid^19^, ADAPTS exhibits extremely stable liquid–liquid interfaces inside the microchannel, due to the ability not only to temporarily store the functional liquid in the air pockets but also to retain a finite amount of the functional liquid at the corners^20^ and on the replenishable porous surfaces of the microchannel^21^ (Fig. 3a, b). Confocal images clearly show a cylindrical shape of the transport liquid inside a square-shaped microchannel and the retention of the functional liquid at the channel corners that is stable even at high flow rates (Fig. 3a). This remaining functional liquid plays a role of a physical barrier separating the solid matrix from the transport liquid (Fig. 3b). The thickness of functional liquid layer reduces with time due to dragging by the transport liquid, and we can model this effect as follows^21–23^:

1 $$h = \left[ {\frac{{0.0003 + 40{\mathrm{Ca}}^2}}{{4D{\mathrm{Ca}}^2}} + 0.295\frac{{64}}{\mathrm{Re}}\frac{{\rho _{\mathrm{F}}}}{{\mu _{\mathrm{F}}L}}\left( {\frac{{4Q}}{{\pi D^2}}} \right)^2t} \right]^{ - 1}$$

where *h* is the thickness of functional liquid layer, *t* is the time, *Q* is the volumetric flow rate, *L* is the channel length, *D* is the channel size, Ca is the Capillary number 4*Qµ*_F_*/πD*^2^, Re is the Reynolds number 4*Qρ*_T_*/πDµ*_T_, *ρ* is the density, and *μ* is the viscosity (Supplementary Table 2). This equation holds when the thickness of the functional liquid layer is much smaller than the channel size (e.g., *h*«*D*), which is consistent with our experimental observation. It allows us to estimate the behavior of the liquid–liquid interface by tuning geometric factors and material properties of the system: the retention of the functional liquid increases with decreasing channel size (Fig. 3c) and with increasing viscosity of the functional liquid (Fig. 3d). Our experiments show that the thickness of the functional liquid has very small variance along the flow direction, and the theoretical prediction agrees well with our experimental results (Fig. 3e). The residual functional liquid layer is sheared by the transport liquid, leading to the reduced thickness of the functional liquid layer, which moves into the air pockets of the partially filled porous matrix. Figure 3b further demonstrates that, besides the retention of functional liquid inside the channel, the porous ADAPTS matrix provides an additional holding mechanism for the displaced functional liquid, which is reversibly transported and stored in the air pockets via capillary pressure (Supplementary Fig. 5). As a result, the ADAPTS possesses a stable functional liquid interface, which serves as a physical barrier to separate the transport liquid from the solid matrix, as well as a mechanism to refill the microchannel with the functional liquid.

### Antifouling property

**Fig. 4:**
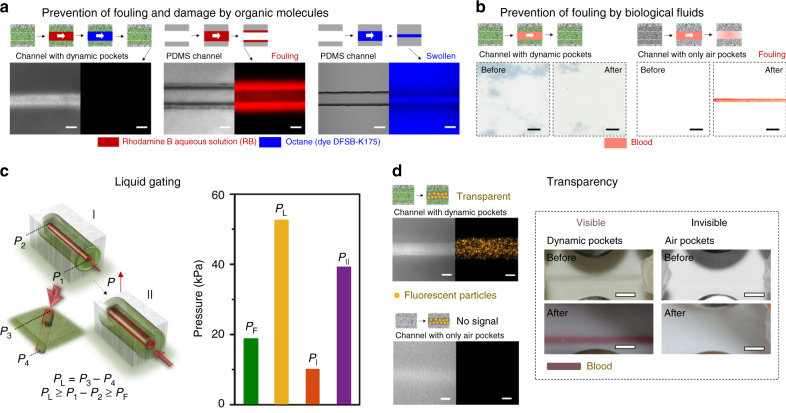
Multifunctional microfluidics enabled by ADAPTS. **a** Antifouling capability of a microchannel with dynamic pockets before and after continuously transporting first RB and then octane. No fouling or soaking of the channel was observed. Conventional PDMS microchannels were injected with RB and octane at the same conditions. A significant amount of residual RB is left on the walls of PDMS microchannels. Octane swells PDMS and damages the entire microchannel. Scale bar is 100 μm. **b** Antifouling capability of a microchannel with dynamic pockets before and after continuously injecting blood. No fouling or soaking of the channel was observed. A liquid-free PTFE microchannel was injected with blood at the same conditions. A significant amount of residual blood is left on the walls of PTFE microchannel. Scale bar is 2 mm. **c** Liquid gating function of the microchannel inside the ADAPTS. The results are shown for the channel size of the length 6 cm, height 200 µm, width 5 mm; average pore size of ~5 µm and flow rate of 500 µL/min (I) and 2.5 mL/min (II). **d** Fluorescent particles inside the microchannel with dynamic pockets show fluorescent signal, while the signals are blocked by the channel with air pockets only. Scale bar is 100 μm. Optical images of blood transport inside the microchannels with or without dynamic pockets indicates that the blood flow is visible inside the ADAPTS. Scale bar is 2.5 mm

The presence of the stable liquid lining gives rise to an exceptional antifouling characteristic. To demonstrate this property, we compare ADAPTS with commonly used PDMS and liquid-free PTFE microchannels in their ability to transport flows of aqueous solution of Rhodamine B (RB), octane, and blood. These transport liquids were chosen as representative examples to test the antifouling properties, since RB is a highly sticky organic molecule that attaches to surfaces through non-specific binding, octane is a good solvent for commonly used polymeric materials that often causes damage of the channel walls, and blood is the most ubiquitous and challenging biological fluid known to indiscriminately adhere to nearly any material. Continuous flows of these fluids show no detectable fouling of the microchannel with ADAPTS (Fig. 4a, b), even after a long-term operation (Supplementary Fig. 6). In contrast, the widely used PDMS is contaminated by the RB (Fig. 4a) and swollen by octane (Fig. 4a). Though considered as a highly non-adhesive material, traditional dry PTFE microchannel that contains only air-filled pores is significantly contaminated by blood (Fig. 4b).

### Multifunctional microfluidics

Besides antifouling, self-recovery, and ability to operate at a wide pressure range described above, ADAPTS also provides other advantageous features, such as liquid gating and transparency. The functional liquid inside the ADAPTS microchannel acts as a dynamic liquid gate that controls the entrance of the transport fluid into and its flow through the channel, in the range of working pressures between *P*_F_ and *P*_L_ (Fig. 4c and Supplementary Fig. 7). *P*_F_ refers to the applied pressure required to initiate a flow pathway of the transport liquid at a certain flow rate (500 µL/min in Supplementary Fig. 7a). *P*_L_ refers to the maximum pressure at which ADAPTS can retain the flow of transport liquid inside the microchannel (Supplementary Fig. 7b). In addition, for active liquid gating, the critical pressures are tunable by controlling the air pocket pressure (*P*_A_) (Supplementary Fig. 8). Moreover, due to the scattering of light within air-filled pockets of PTFE, microchannels made of dry PTFE are non-transparent, making them unsuitable for observing the flow in the system. In contrast, high transparency of the ADAPTS microchannels can be obtained by tuning the refractive index of the functional liquid to that of the surrounding porous matrix, which enables an easy optical monitoring and detection of fluorescent or colored materials (Fig. 4d; Supplementary Fig. 9; Supplementary Movie 1).

### Broad design and material selection

The design of ADAPTS is readily extended to other material systems that can offer additional practical advantages. While polymers are widely used to form microfluidic networks and membranes, their limited thermal and mechanical stability prevents their applications at high pressures or temperatures. To address these issues and further enrich the materials options for the ADAPTS design, we have developed metal-based porous materials (Supplementary Fig. 10)^24^. A chemically modified single-layer porous stainless steel was used to achieve highly stable flow properties and the ability to operate at significantly higher temperature and flow rate compared to those made of polymers (Supplementary Fig. 11a-c). Such microchannel structures can, therefore, offer a simple and universal solution for antifouling within a stable fluidic network and be applied to a variety of microchannels within a functionalized solid matrix, infused with a low-surface-tension liquid. The latter is retained in place due to its ability to move to and from the air pockets, thus providing a stable “antifouling” liquid interface that can dynamically guide microscale flows (Supplementary Fig. 11d).

## Discussion

**Fig. 5:**
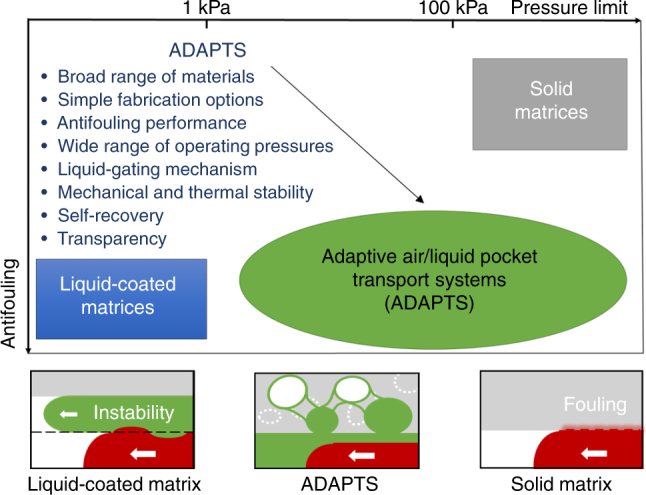
Qualitative comparison in terms of antifouling and operating pressure. Schematics illustrate the surface fouling and instability associated with the solid matrix and liquid-coated matrix, respectively

To conclude, we have theoretically and experimentally demonstrated a dynamic air/liquid pocket system for controlling microscale flow. The multifunctionality and advantages of the ADAPTS microfluidics, which may benefit many practical applications^25^, are summarized in Fig. 5. The design of ADAPTS breaks the long-lasting restrictions in microscale flow regulation, by simultaneously providing a stable, reversible liquid–liquid interface that enables exceptional antifouling properties against even the most tenacious contaminants such as biological fluids, and a robust solid matrix that can sustain high temperature and pressure. The ease of fabrication of ADAPTS from various materials, such as diverse commercially available porous matrices and functional liquids, holds promise for mass production. As the research on microscale flow regulation continues to push the limits, the design of ADAPTS would be highly appealing for the in situ formulation of inks for advanced printing^7^, biomedical microfluidics, lab-on-a-chip platforms^26^, and surface and membrane technologies such as liquid-gated and nanocrack-regulated membranes^27,28^. The dynamic air/liquid pockets described here and the broader applications of ADAPTS design open up opportunities to dynamically regulate microscale flows in many areas ranging from drug delivery and microscale reactors for production of high-value fluids to soft robotics, microfluidic computational devices^29^, medical devices, and microchip sensors for bioassays and environmental analysis^30–33^.

## Methods

### Porous membranes

Three types of Teflon membranes were purchased from Sterlitech Corporation, WA, USA, with the average pore sizes of ≥20 µm (thickness of ~150 µm), ≥5 µm (thickness of ~200 µm), and ≥200 nm (thickness of ~30 µm). The membranes with the average pore size of ≥5 µm were evaluated by scanning electron microscopy (Supplementary Fig. 2a). Thin membranes with the average pore size of ≥200 nm were used for the confocal experiments to visualize the liquid transport inside the three-dimensional (3D) microporous matrix.

### PDMS channels

Sylgard 184 silicone elastomer base and Sylgard 184 silicone elastomer curing agent were purchased from Dow Corning Corporation. PDMS mixed at a 10:1 curing ratio was placed into microchannel molds and cured for 3 h at 70 °C.

### Functional liquids

Krytox®103 (purchased from DuPont (USA) and silicone oil (purchased from www.sigmaaldrich.com (481939 - Poly(dimethylsiloxane)) were used as functional liquids.

### Preparation of the 3D microporous matrix

First, the high-resolution channel pattern and 3D arrangement was designed using various suitable software packages. Versalaser cutting engraving system (CNS, Harvard University) was then used to cut the channels or ports on the membrane directly (Supplementary Fig. 2b-d). It is worth mentioning that the dimensions of the microchannels are limited by the source of the porous membranes and the fabrication technique. Besides the PTFE membrane and the laser cutting technique used in this study, the design of ADAPTS can be realized with many other porous membranes and fabrication approaches, including blade cutting, photolithography, 3D printing, and thermo-molding technique, and can be therefore easily achieved in various laboratories without using clean rooms or involving expensive equipment and harsh chemicals. Second, transparent PMMA sheets and stainless-steel screws were used to seal the microchannels inside the microporous matrix to avoid the leaking problem. Third, the functional liquid was infused into the microchannels using a fluid delivery syringe pump (Harvard Apparatus’ PHD ULTRA CP Syringe Pump) equipped with syringes (NORM-JECT). With our approach, various complex microchannel shapes can be generated (Supplementary Fig. 2e) and further arranged in a multi-layered system by 3D stacking layers method (Supplementary Fig. 3), which makes it ideal for a variety of specific applications and amenable for mass production.

### Test fluids

The test fluids, which include octane (puriss, ≥99.0%) and RB (high-performance liquid chromatography, ≥97.0%), were obtained from Sigma Aldrich. Sheep blood in heparin (3 IU/mL) was obtained from HemoStat Laboratories, CA, USA. RB aqueous solution (RB): RB was dissolved in deionized water (DI) to give the RB solution at a final concentration of 0.1 mg/mL. Dye DFSB-K175 (from www.riskreactor.com) was dissolved in octane with 0.10 Vol%. The antifouling properties have been studied by infusing RB (15 min), octane (15 min), and sheep whole blood (7 h) with the same flow rate of 10 μL/min at 20 °C.

### Fluorescent measurements

Zeiss Confocal Laser Scanning Microscope from Carl Zeiss Microscopy GmbH, Jena, Germany, (LSM 700) was used for fluorescent and confocal experiments. For dye RB, the measuring parameters were automatically set up from the database of Zeiss microscopy system. DFSB-K175 was detected for a broad wavelength range (≥560 nm) and laser line (488 nm). The fluorescent particles were detected for a broad wavelength range (≥500 nm) and laser line (488 nm). DI water with a resistivity of 18.2 MΩ·cm was used for the measurements. Microparticles in the suspension were the surfactant-free fluorescent yellow green sulfate latex with the diameter of ~1.6 μm (Solid%: 1.9) obtained from Invitrogen. Fluorescent particles suspension was made using 0.1 mL of the original 1.9% suspension diluted in 2 mL of H_2_O, to approximately 0.10 Vol%. The functional liquid used for confocal images was the silicone oil with dye DFSB-K175.

### Data availability

The authors declare that the data supporting the findings of this study are available within this paper and its Supplementary Information file or from the corresponding author.

## Electronic supplementary material

Supplementary Information

Description of Additional Supplementary Files

Supplementary Movie 1
