# Supplementary material for: Dynamic air/liquid pockets for guiding microscale flow
Source: Nat Commun. 2018 Feb 21;9:733. doi: 10.1038/s41467-018-03194-z (PMC5821814; doi:10.1038/s41467-018-03194-z)
Supplement: Supplementary file 1 — Supplementary Information [file 41467_2018_3194_MOESM1_ESM.pdf]

Supplementary Information

Dynamic Air/Liquid Pockets for Guiding Microscale Flow

Hou *et al.*

Supplementary Note 1: Bio-inspired Design Concept.

Our design is inspired by the mucus lining in colon and stomach that separate gastrointestinal fluids and microbes from the epithelium [1]. These surfaces have evolved into micro-porous structured layers infiltrated with a protective liquid mucus, secreted by mucous cells underneath and continuously supplied to the epithelium (**Supplementary Figure 1a**). Our artificial system, ADAPTS, not only has the protective liquid that is supplied from the porous matrix to the interface but also has air pockets in its partially-infiltrated walls, which can absorb and store the functional liquid displaced by the flow in the microchannel, thus offering unique dynamic, reversible properties (**Supplementary Figure 1b**).

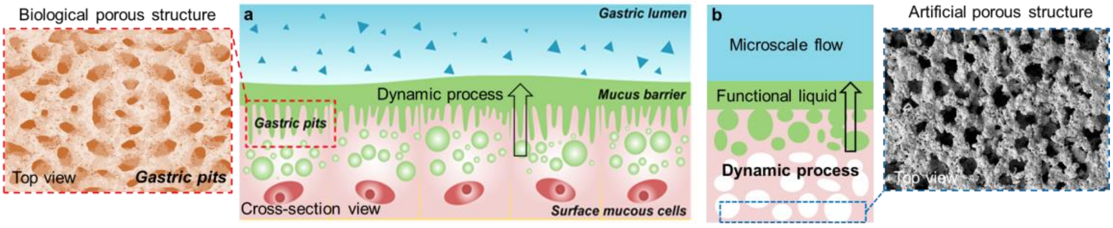

Supplementary Figure 1 | Design of the ADAPS system inspired by the structure of the surfaces in the gastrointestinal tract. a Diagram of mucosal defense mechanism in stomach. The "hole-like", microporous nature of the gastric pits in the gastric lumen (shown in the inset), and cardiac glands provide mucous secretion to coat the epithelium and protect it from erosion in the harsh environment of the stomach. An intriguing mechanism is that those surfaces have evolved into micro-porous structured layer infiltrated with a protective liquid mucus, and with continuous supply of mucus secreted by mucous cells underneath. Schematic adapted from gastric mucosal barrier at https://en.wikipedia.org/ and stomach lining with gastric pits at https://fineartamerica.com/. **b** The bioinspired design of dynamic, stable functional liquid layer to protect the microchannel surface of ADAPTS. The inset shows a SEM image of an artificial porous matrix (a copper porous membrane with the average pore size ~50 μm).

Supplementary Methods：

Materials

PTFE porous membranes for adaptive air/liquid pocket transport system: Three types of Teflon membranes were purchased from Sterlitech Corporation, WA, USA: the average pore sizes of ⩾ 20 μm (thickness of ~150 μm), ⩾ 5 μm (thickness of ~200 μm), and ⩾ 200 nm (thickness of ~30 μm). The membranes with the average pore size of ⩾ 5 μm were evaluated by scanning electron microscopy (SEM) (**Supplementary Figure 2a**). Thin membranes with the average pore size of ⩾ 200 nm were used for the confocal experiments to visualize the liquid transport inside the 3D microporous matrix.

Polydimethylsiloxane (PDMS) channels: Sylgard 184 silicone elastomer base and Sylgard 184 silicone elastomer curing agent were purchased from Dow Corning corporation. PDMS mixed at a 10:1 curing ratio is placed into microchannel molds and cured for 3 hours at 70°C.

Polymethylmethacrylate (PMMA) sheets: 1/16" thick and 3/16" thick scratch-resistant clear cast acrylic 12" X 12" sheets were purchased from McMaster-Carr Supply Company.

Functional liquids: The functional liquids used in the experiments were Krytox®103 and silicone oil. Unless otherwise specified, Krytox®103 was used throughout the antifouling experiments. DuPont Krytox® GPL K103 was purchased from DuPont (USA). The silicone oil was purchased from www.sigmaaldrich.com (481939 - Poly(dimethylsiloxane). Dye DFSB-K175 was obtained from www.riskreactor.com. The silicone oil with dye DFSB-K175 was used for 3D confocal imaging. Deionized water (DI water) with a resistivity of 18.2 MΩ·cm was used for the measurements.

Test fluids included:

> *Rhodamine B aqueous solution (RB)*: Rhodamine B (HPLC, ⩾ 97.0%, obtained from Sigma Aldrich) was dissolved in DI water at a final concentration of 0.1 mg/mL.
>
> *Fluorescently labelled organic liquid*: Dye DFSB-K175 was dissolved in octane (puriss, ⩾ 99.0%, obtained from Sigma Aldrich) at 0.10% (w/v).
>
> *Biological fluids*: Sheep blood in heparin (3 IU/mL) was obtained from HemoStat Laboratories, CA, USA.
>
> *Fluorescently labelled microparticle suspension*: Surfactant-free fluorescent latex beads were purchased from Invitrogen (diameter of ~1.6 μm; solid content of 1.9 %). The stock solution was diluted with DI water at a volume ratio of 1:20 to get 0.10 Vol% suspension used in the experiments.

Artificial porous matrix with porous surface similar to the gastric pits: Cu porous membranes were purchased from Soochow Jiashide Corporation, China. The average pore size is 50 μm. The thickness is ~500 μm. The membranes with the average pore size of 50 μm were evaluated by SEM (**Supplementary Figure 1b,** inset).

Metal-based porous membranes: Sodium tungstate dihydrate, nickel chloride, boric acid, cobalt acetate were purchased from Sigma-Aldrich (USA). Potassium nitrate was purchased from J.T.Baker (USA). MilliQ DI water was used in all experiments; acetone and ethanol (200-Proof) were purchased from VWR. AISI 304 and 316 grade stainless steel foils were purchased from McMaster-Carr.

Preparation of the ADAPTS microchannel

The ADAPTS microchannel is fabricated in 3 steps. First, computer-aided design was conducted to make high-resolution channel patterns and 3D arrangement. Versalaser cutting engraving system (CNS, Harvard University) was then used to cut the channels or ports on the membrane (**Supplementary Figure 2b-2d**). It is worth mentioning that the preparation of the microchannels inside the 3D microporous matrix is compatible with various other technologies to make all kinds of shapes of channels besides laser cutting, such as blade cutting, photolithography, and thermo-molding technique, and can be therefore easily conducted in laboratories without clean rooms or sophisticated equipment. Second, transparent PMMA sheets and stainless-steel screws were used to seal the microchannels inside the microporous matrix to prevent leakage. Third, the functional liquid was infused into the microchannels using a fluid delivery syringe pump (Harvard Apparatus' PHD ULTRA CP Syringe Pump) equipped

with syringes (NORM-JECT). With our approach, various complex microchannel shapes can be generated (**Supplementary Figure 2e**), and further arranged in a multi-layered system by 3D stacking layers method (**Supplementary Figure 3**), which makes it adaptable for a variety of specific applications and suitable for mass production.

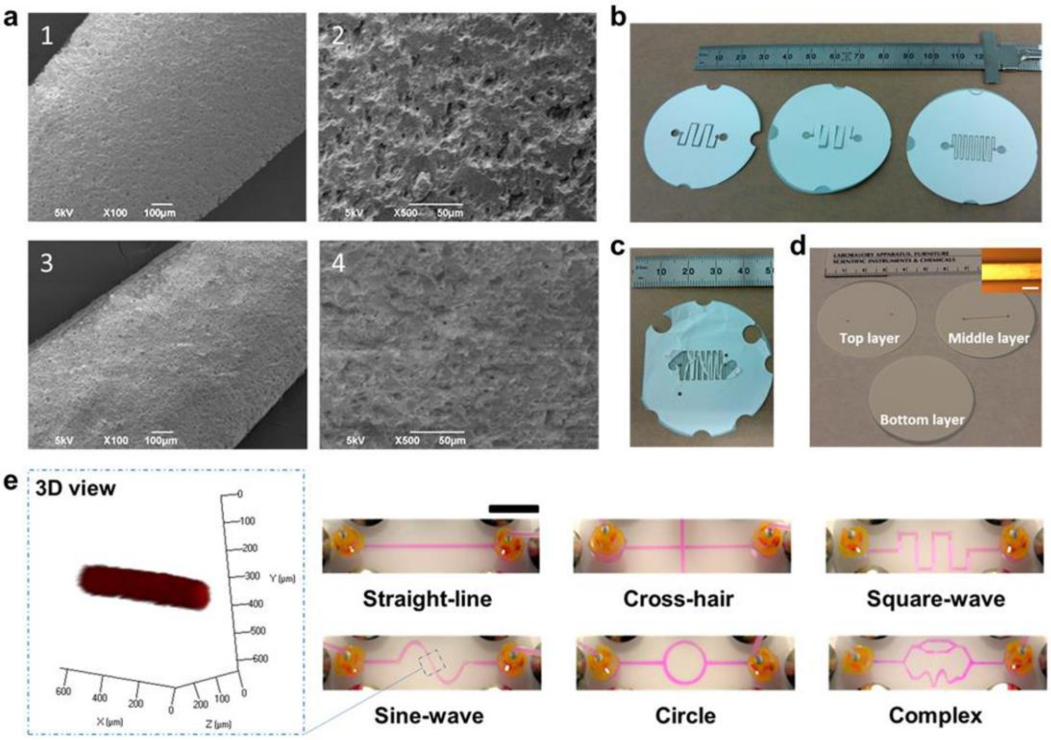

Supplementary Figure 2 | Materials preparation. a Scanning electron microscope images of the PTFE porous membrane with average pore size ≥ 5 µm: Top surface (**1-2**); Bottom surface (**3-4**). Digital photos of the porous membrane with constructed microchannels resulted from an optimal laser cutting conditions (**b**) and from suboptimal conditions (**c**). **d** Digital photos of 3 layers used to assemble the ADAPTS microchannel. The inset shows a micrograph of the microchannel (scale bar 300 µm). **e** Digital images of the microchannels of various shapes which transport the Rhodamine B (RB) aqueous solution inside (scale bar 1 cm). The inset on the left shows the confocal image of the flow pathway of the RB solution.

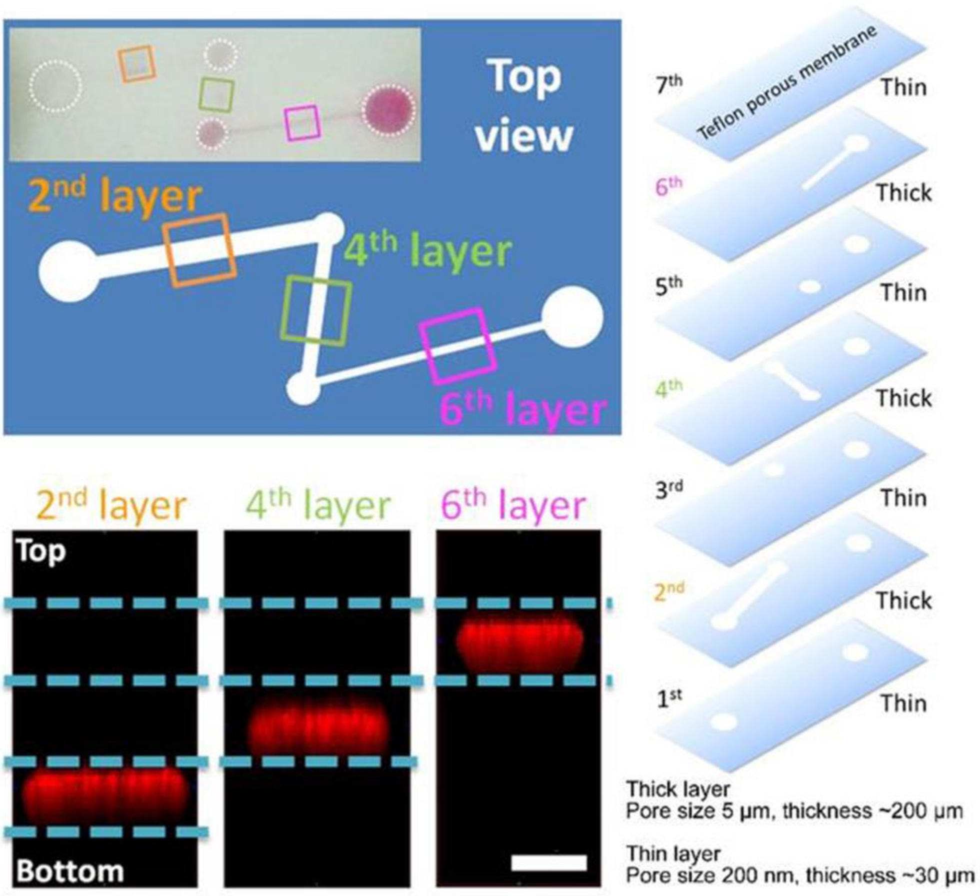

Supplementary Figure 3 | Multi-layered assembly of ADAPTS microchannels. Schematic of the design for multi-layered microchannels (Top left, and right). The confocal images of multi-layered microchannels visualized by infusing Rhodamine B aqueous solution (Bottom left). Scale bar is 200 μm.

Contact angle measurements

The contact angle measurements were performed using KSV CAM 101 system at room temperature (i.e., 20 – 24°C) with ~20% relative humidity. The system was calibrated before all the measurements were taken.

Supplementary Table 1. Measured interfacial tension for various liquids.

Liquid	Interfacial tension (mN/m)		Volume (μL)	
	Mean	SD	Mean	SD
DI-Water	72.05	1.20	12.79	0.21
DI-Water (dye RB)	63.77	0.50	11.44	0.14
Krytox®103	17.55	0.31	1.66	0.06
DI-Water/Krytox®103	56.76	1.21	11.27	0.25
DI-Water (dye RB)/Krytox®103	40.59	0.35	7.86	0.06
Silicone oil/water	24.35	0.34	[2]	

Supplementary Table 2. Dynamic viscosity of various liquids.

Liquid	Dynamic viscosity (Pa·s)	References
Krytox®103	~ 0.14208 (at 20°C)	http://www.matweb.com/
H_20	0.001002 ~ 0.0007978 (at 20 ~ 30°C)	http://en.wikipedia.org/wiki/Viscosity
Silicone oil	~0.025 (at 25°C)	http://www.sigmaaldrich.com/united-states.html

Fluorescent measurements

Zeiss Confocal Laser Scanning Microscope from Carl Zeiss Microscopy GmbH, Jena, Germany, (LSM 700) was used for fluorescent and confocal experiments. For dye RB, the measuring parameters were automatically set up from the database of Zeiss microscopy system. The dye DFSB-K175 was detected for a broad wavelength range (⩾ 560 nm), and laser line (488 nm). The fluorescent particles were detected for a broad wavelength range (⩾ 500 nm), and laser line (488 nm). Deionized water with a resistivity of 18.2 MΩ·cm was used for the measurements. Microparticles in the suspension were the surfactant-free fluorescent yellow green sulfate latex with the diameter of ~1.6 μm (Solid%: 1.9) obtained from Invitrogen. Fluorescent particles suspension was made using 0.1 mL of the original 1.9% suspension diluted in 2 mL of H_2O, to approximately 0.10 Vol%. The functional liquid used for confocal images was the silicone oil with dye DFSB-K175.

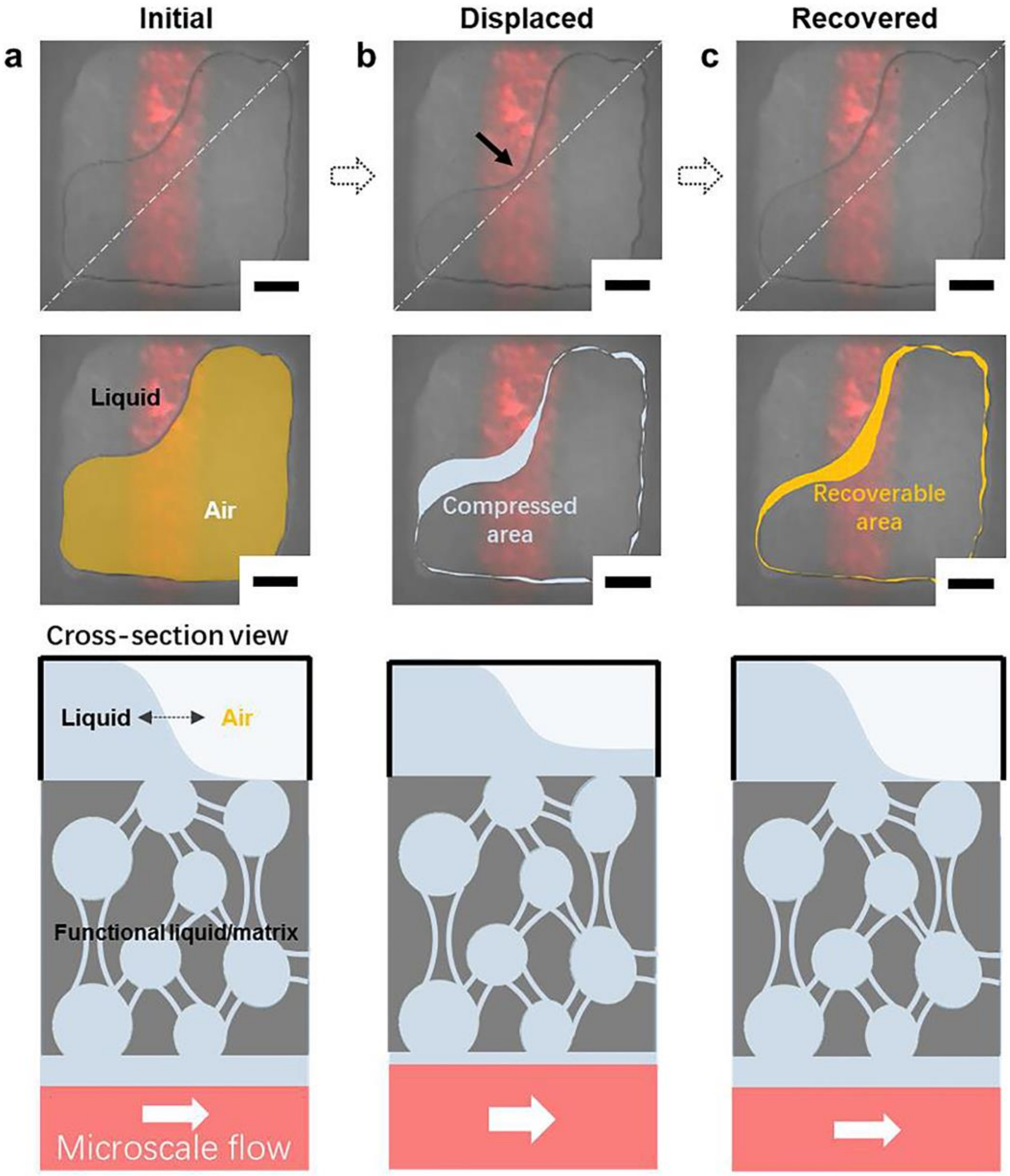

Supplementary Figure 4 | Forced displacement and self-recovery of the functional liquid. a A large air pocket is constructed on top of an ADAPTS microchannel, which transports the RB solution. The changes in the shape and size of this air pocket (highlighted in yellow) provide the evidence for the movement of the functional liquid to and from the porous matrix. Scale bar is 200 μm. **b** The large air pocket shrinks as the functional liquid is driven by the applied pressure out of the liquid pockets. The amount of shrinkage is marked in light blue. Scale bar is 200 μm. **c** When the pressure is removed, the large air pocket drives the displaced functional liquid back into the ADAPTS matrix, resulting in restoration of the system. The area change is marked in yellow. Scale bar is 200 μm.

Dynamic movement of the functional liquid in ADAPTS

Displacement of the functional liquid by the transport liquid: A square-shaped microchannel of width D is constructed inside an ADAPTS matrix of average pore size ξ, and filled with a functional liquid in the initial state. The functional liquid is displaced by a transport liquid, resulting in a cylindrical flow pathway. The liquid-liquid displacement takes place only if the applied pressure (ΔP) is equal to or greater

than a threshold. It is a critical pressure (P_0) that relates to the channel size D and the liquid-liquid interfacial tension $\boldsymbol{\gamma}_{FT}$ via $4\boldsymbol{\gamma}_{FT}/D$.

The design of ADAPTS can retain a finite amount of functional liquid in the microchannel even under high flow rates. Due to the preferential wettability of the porous matrix with the functional liquid and to the non-circular shape of the microchannel, the channel corners hold the functional liquid. In addition, the inner surface of the microchannel retains a thin layer of the functional liquid. Such phenomenon has been investigated in the context of the pressure-driven core-annular flow. The residual functional liquid is formed after the liquid-liquid displacement and then further sheared out by the transport liquid over time due to the dragging force. The thickness of the residual functional liquid evolves with time via

$$h = \left[\frac{1}{H} + 0.295\frac{64}{Re}\frac{\rho_F}{\mu_F L}\left(\frac{4Q}{\pi D^2}\right)^2 t\right]^{-1} \tag{1}$$

where Q is the volumetric flow rate, L is the channel length, D is the channel size, Re is the Reynolds number $4Q\rho_T/\pi D\mu_T$, ρ is the density, μ is the viscosity, and the subscripts F and T denote the functional liquid and the transport liquid, respectively, and H is the initial thickness of the residual functional liquid after the displacement, which is governed by the capillary number $Ca = 4Q\mu_F/\pi D^2\boldsymbol{\gamma}_{FT}$ via

$$H = \frac{4Ca^2}{0.0003+40Ca^2}D \tag{2}$$

Movement of functional liquid inside the porous matrix: When a transport fluid flows along the microchannel direction, the functional liquid is driven by the pressure to move along the directions perpendicular to the microchannel direction (**Supplementary Figure 5a**), and infiltrate into the porous matrix. The volumetric flow rate of the functional liquid heading into the porous media Q_{LA} relates to the applied pressure ΔP (**Supplementary Figure 5b**) via Darcy's law:

$$Q_{LA} = \frac{kA\Delta P}{\mu_F L} \tag{3}$$

where A is the inner surface area of the microchannel, k is the permeability of the porous media (typically on the order of 10^{-14} m^2), μ_F is the viscosity of the functional liquid (0.142 Pa·s), L is the length of the microchannel (**Supplementary Figure 5c**). The relation holds until the functional liquid is depleted inside the microchannel, or the transport liquid invades the porous matrix if the pressure exceeds the limit pressure (P_L).

Movement of functional liquid from microchannel into air pockets: The air pockets absorb the functional liquid from the microchannel due to the capillary effect. The phenomenon is known as wicking process, which is governed by the viscous effect and the capillary force. Considering the small radius of microscale pores, the inertia effect is negligible. The air pockets can be simplified as micro-tubes of the average pore size ξ. The wicking depth of the functional liquid into the air pockets follows the Washburn's law:

$$x(t) = \frac{\xi}{4}\frac{\gamma cos\theta}{\eta}t \tag{4}$$

where θ and $\boldsymbol{\gamma}$ denote the contact angle and the surface energy of the functional liquid-solid matrix interface; in the case of the lubricant-PTFE system, $\boldsymbol{\gamma} = 3\ mN/m$

and $\theta = 0^o$. **Supplementary Figure 5d** plots the wicking process: the channel size D = 200 μm, and the porosity of PTFE matrix p = 50%, the average pore size ξ=5 μm. By the volume conservation, we have

$$\pi\left(\frac{D}{2}\right)^2 = \left[\pi\left(\frac{d}{2}\right)^2 - \pi\left(\frac{D}{2}\right)^2\right] \times p \tag{5}$$

where d is the size of the region where the functional liquid wicks. From the relation, we can estimate the maximum penetration depth of the lubricant that initially is in the channel:

$$x_{max} = \frac{1}{2}D\left(\sqrt{1+\frac{1}{p}} - 1\right) \tag{6}$$

Therefore, the time required to deplete the functional liquid in the microchannel is estimated by

$$t_{req} = [\frac{1}{2}\frac{D}{q}(\sqrt{1+\frac{1}{p}} - 1)]^2 \tag{7}$$

where $q = \sqrt{\frac{1}{4}\frac{\gamma\xi cos\theta}{\eta}}$. With those physical parameters, the required time for wicking is estimated to be around 0.2 s, indicating a rapid infusion process that can respond almost immediately to retain the displaced functional liquid (**Supplementary Figure 5d**). From the above relationships, we can also calculate the thickness of the matrix of air pockets required to retain all the functional liquid displaced from the microchannel, $H_A = t_{req} \times V$, where V is the flow rate (**Supplementary Figure 5e**), which is evidently independent of the channel size.

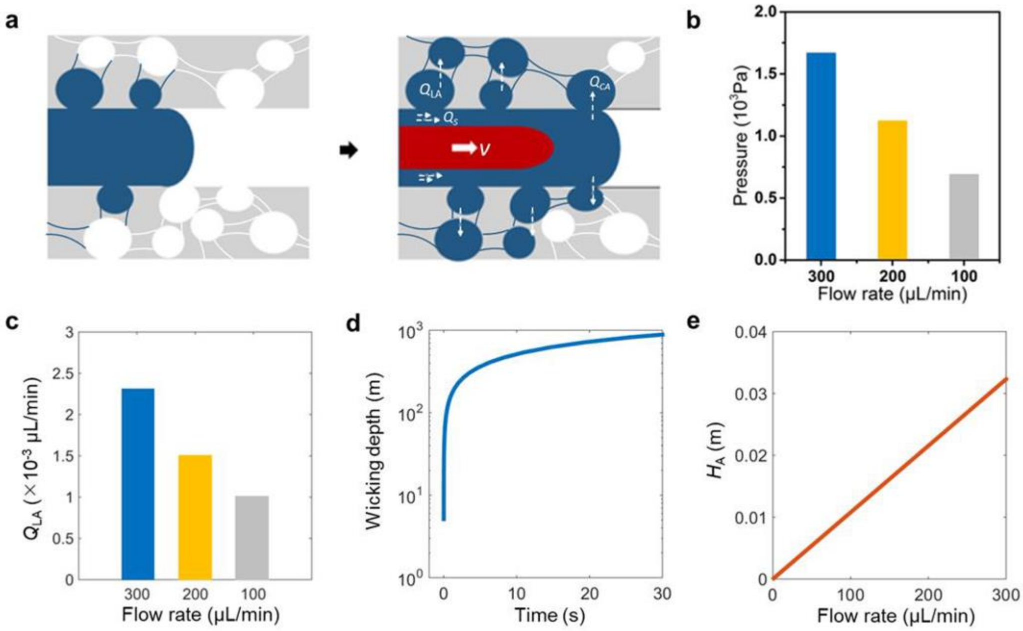

Supplementary Figure 5 | Movement of functional liquid in ADAPTS. **a** Schematics show infiltration of the functional liquid (blue) in the porous matrix (grey) before and after the transport liquid (red) invades the microchannel. The volumetric flow rate of the functional liquid moving from the microchannel to the air pockets is denoted as Q_{CA}, and that from the liquid pockets to the air pockets as Q_{LA}. **b** Measured pressures in the microchannel as a function of the flow rates. **c** Calculated volumetric flow rate (Q_{LA}) of the functional liquid moving between the liquid pockets and the air pockets. **d** Wicking depth of the functional liquid moving from the microchannel into the air pockets. **e** Required thickness of the matrix with air pockets to retain the displaced functional liquid as function of the flow rate.

Antifouling performance

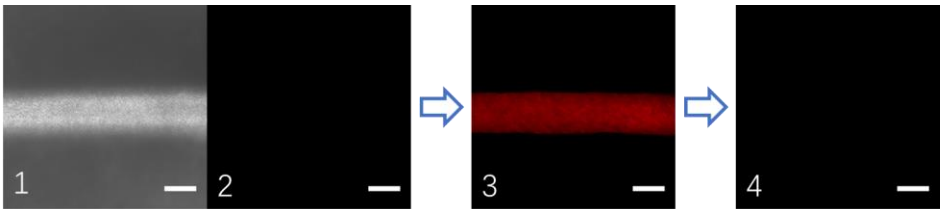

Supplementary Figure S6 | Fouling after one-hour operation. Optical image (1) and fluorescence image (2) of the microchannel before infusing Rhodamine B aqueous solution. (3), after infusing Rhodamine B aqueous solution at the rate of 10 μL/min for 1 hour. (4), after infusing air at 10 μL/min. Scale bar is 100 μm.

Liquid-gating properties

The functional liquid in the ADAPTS gates the entrance of the transport liquid. **Supplementary Figure 7** shows the flow of the transport liquid in the liquid-gated channel within the working pressure interval from P_F to P_L. The minimum working pressure P_F is the critical pressure of water determined by transporting the DI water through the microchannel at the flow rate of 500 μL/min (**Supplementary Figure 7a**). The maximum working pressure P_L is the critical pressure of water determined by transporting DI water through the ADAPTS at the flow rate of 2500 μL/min (**Supplementary Figure 7b**).

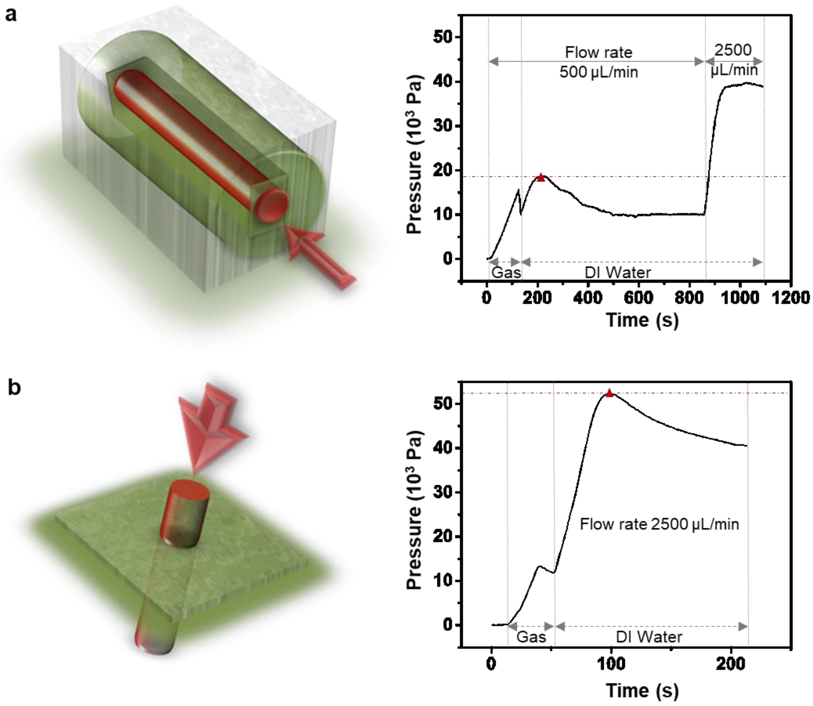

Supplementary Figure 7 | Liquid gating microchannel inside the ADAPTS. a Critical pressure of DI water flowing through the microchannel. Channel size: height 6 cm, length 200 μm, width 5 mm; pore size ~5 μm. **b** Critical pressure of water flowing inside ADAPTS.

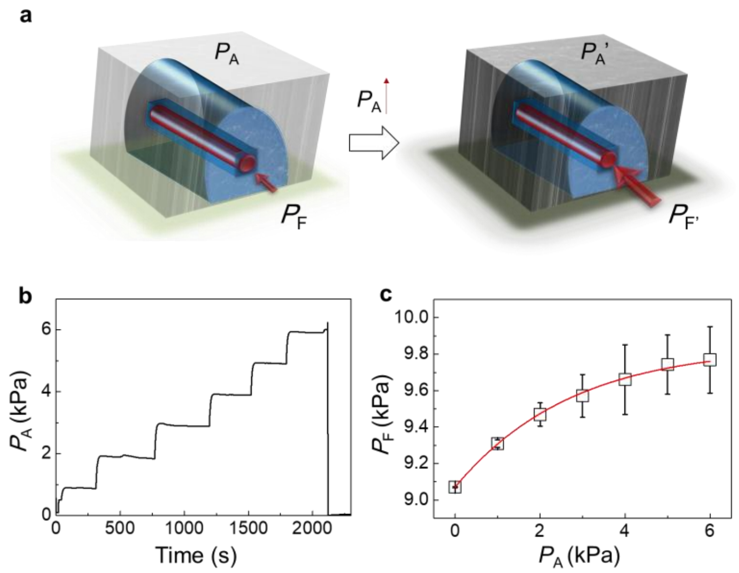

Supplementary Figure 8 | ADAPTS with active control over critical pressures. a Schematic showing that the pressure in the air pockets (P_A) surrounding the functional liquid is controlled by an external pressure pump. The critical pressure for forming a flow path inside the microchannel (P_F) is recorded. As P_A increases, different values of P_F are obtained. The channel size: length 10 mm, height 200 µm, width 100 µm; the pore size of ~5 µm; the flow rate 1000 µL/min for the transport DI water. **b** The pressure in the air pockets (P_A) was increased over time in a step-wise fashion. **c** The measured critical pressure P_F is plotted as a function of the applied pressure in the air pockets P_A. The red line is used to highlight the trend. Error bars show standard deviation. Sample number N=3.

Transparency of the ADAPTS

The ADAPTS can be rendered transparent by matching the refractive index of the functional liquid to that of the matrix. This allows the easy monitoring of the liquid transport in the channel, which is impossible in traditional, non-transparent air-filled PTFE channels (**Supplementary Figure 9**).

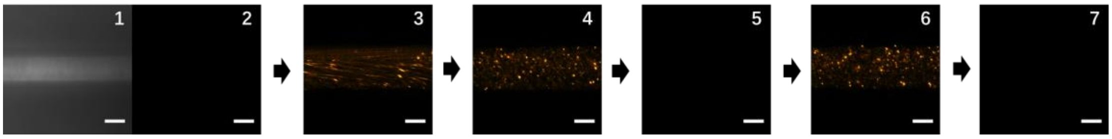

Supplementary Figure 9 | Repetitive On/Off operation of ADAPTS enabled by the transparency of the functional liquid-infused microchannels. Optical image (1) and fluorescent image (2) of the microchannel at the beginning. Fluorescent images of the microchannel (3) when *running* a solution of fluorescent particles (yellow) at a flow rate of 10 µL/min, (4) when the applied pressure was kept at the threshold value and the flow stopped, (5) after removal of the applied *pressure* and return of the functional liquid from the porous network to the microchannel, (6) when the applied pressure was kept at the threshold value and the flow stopped after 15 cycles of on/off switching operation, (7) after removal of the applied pressure and return of the functional liquid from the porous network to the microchannel. Scale bar is 100 µm.

Preparation of metal-based ADAPTS

The stainless steel membranes were created by electrochemical etching in a standard 50 mL two-electrode-cell using Keithley 2450 SourceMeter® unit. Before etching, the stainless steel foil was cleaned in aqueous detergent (Alcojet, USA) with sonication, rinsed with acetone and DI water, and finally dried with N_2 at room temperature. The etching process was carried out under galvanostatic conditions of constant current density I = 0.2 A/cm^2 with the varied etching time of 10 - 80 s in a freshly-prepared naturally-aerated solution at 60 °C with continuous stirring. 0.5 M H_3BO_3 was added into 2.5 M $NiCl_2$ electrolyte solution as a pH buffer agent. AISI 304 and 316 stainless steel foils with the thickness of 25 - 100 µm (but the same area of 20 × 20 mm^2 for all the samples) were used as an anode and a Cu mesh with similar dimensions was used as a cathode. Then, the electrochemical deposition of porous tungstate membranes was performed according to the previously-reported procedure (Nat. Commun. 2015 6, 8649). Briefly, an aqueous solution of sodium tungstate (0.5 M) was used as the electrolyte. The electrodeposition was performed in a standard three-electrode-cell using potentiostat/galvanostat (Princeton Applied Research, VersaSTAT3-200) and VersaStudio software (Princeton Applied Research). All measurements were carried out at room temperature without stirring or deaeration of the solution. Stainless steel foil was used as the working electrode after cleaning it with aqueous detergent (Alcojet, USA), rinsing with acetone and DI water and drying with N_2 stream at room temperature. The anode was a Pt gauze (10 × 20 mm^2) and the reference electrode was an Ag|AgCl NaCl(sat) (BASi, MF-2052). All potential values are referred regarding to this reference electrode. The cathodic electrochemical deposition was performed at a square waveform pulse potential with a pulse duration of 10 s separated by intervals of 10 s. The voltage of -1.5 V was applied for 12 h. After deposition, samples were removed from the solution, extensively rinsed with DI water and dried with N_2 stream at room temperature. (**Supplementary Figure 10**)

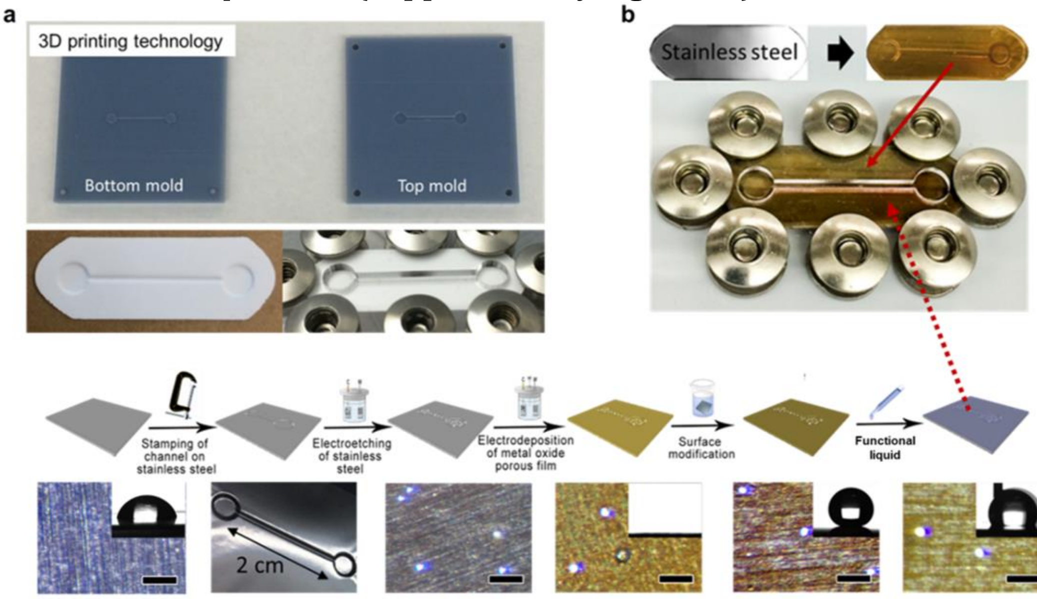

Supplementary Figure 10 | Preparation of polymer-based microchannel (**a**) **and metal-based microchannel of ADAPTS** (**b**). Schematic representation, optical microscopy and water contact angle images of liquid-infused metal-based porous matrix prepared by using electrochemical etching and deposition methods (Bottom). Scale bar is 100 µm.

Compared to the polymer-based material, the metal-based material could achieve a better mold replication property (**Supplementary Figure 11a**). In addition, the metal-based ADAPTS can withstand much higher temperature and higher pressure than polymer-based ADAPTS (**Supplementary Figure 11b, 11c**). It is also worth mentioning that, the metal-based ADAPTS exhibits a sustained antifouling behavior compared with the conventional metal-based microchannel (**Supplementary Figure 11d**).

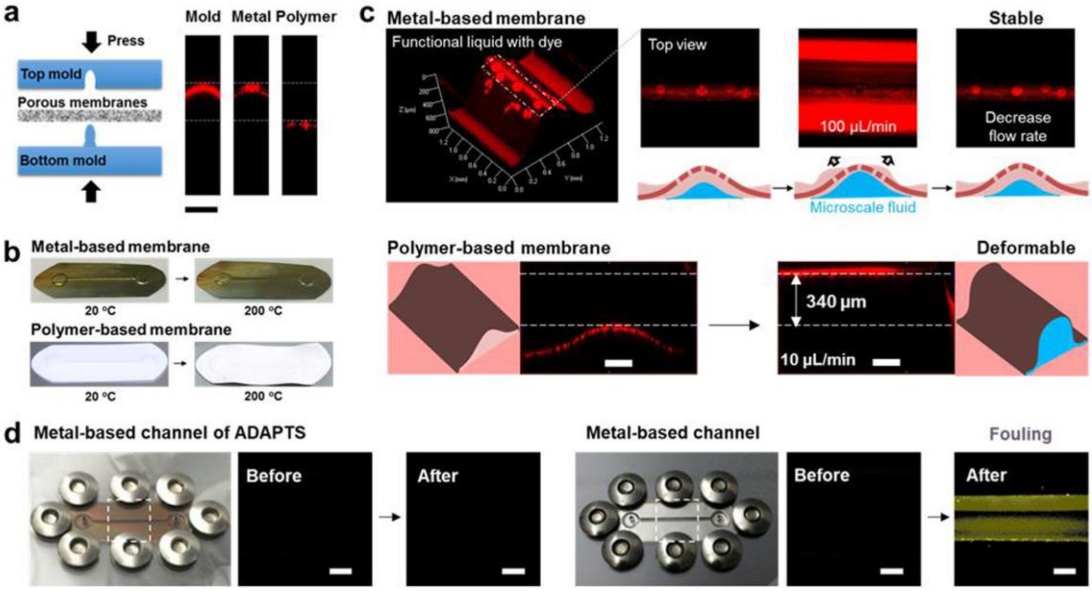

Supplementary Figure 11 | Microscale fluids inside metal-based ADAPTS. a Microchannel of ADAPTS prepared by compression molding. From confocal images, the shape replication property of metal-based materials is much better than polymer-based materials. Scale bar is 0.3 mm. **b**,**c** Exceptional stability under high temperature and high pressure of metal-based ADAPTS. Scale bar is 200 μm. **d** Fluorescent images of metal-based systems before and after injecting RB. Compared with conventional metal-based system, ADAPTS shows excellent antifouling property. Scale bar is 200 μm.

Supplementary References:

[1] M. E. V. Johansson, G. C. Hansson, Keeping bacteria at a distance. *Science*. **334**, 182-183, (2011)

[2] P. Than, L. Preziosi, D. D. Joseh, M. Arney, Measurement of interfacial tension between immiscible liquids with the spinning rod tensiometer. *J. Colloid Interf. Sci.* **124**, 552-559, (1988)
