# Supplementary material for: Dynamic air/liquid pockets for guiding microscale flow
Source: Nat Commun. 2018 Feb 21;9:733. doi: 10.1038/s41467-018-03194-z (PMC5821814; doi:10.1038/s41467-018-03194-z)
Supplement: Supplementary file 2 — Description of Additional Supplementary Files [file 41467_2018_3194_MOESM2_ESM.pdf]

Description of Additional Supplementary Files

File Name: Supplementary Movie 1
Description: A fluorescent particle-containing solution was used to visualize the switchability of the flow in ADAPTS microchannel. When the applied pressure is higher than the threshold value, the fluorescent particle-containing solution would enter the microchannel. When the applied pressure is reduced below the threshold value, the flow is stopped and functional liquid refills the microchannel.
